# Surgical management of aorto-right atrial fistula induced by Stanford type A aortic dissection: a case report

**DOI:** 10.1186/s44215-024-00163-5

**Published:** 2024-08-28

**Authors:** Takahito Yokoyama, Yasutoshi Tsuda, Katsuyuki Shigehara, Ryo Niside, Daiki Sato, Masato Nakajima

**Affiliations:** Department of Cardiovascular Surgery, Yamanashi Prefectural Central Hospital, 1-1-1 Fujimi, Kofu, Yamanashi 400-0027 Japan

**Keywords:** Stanford type A aortic dissection, Aortic-right atrial fistula, Direct closure by suturing

## Abstract

**Background:**

The occurrence of aorto-right atrial fistula in a patient with Stanford type A aortic dissection is exceedingly rare, and the treatment has not been established.

**Case presentation:**

A 60-year-old male presented to the emergency department with acute lumbar pain and, based on contrast-enhanced computed tomography, was diagnosed with Stanford type A aortic dissection. Emergency surgery was performed. Transesophageal echocardiography during the surgery did not reveal an aorto-right atrial fistula. After establishing cardiopulmonary bypass, circulatory arrest was induced, and the primary entry in the proximal ascending aorta for the aortic dissection was identified. The aorta was dissected between the right brachiocephalic artery and the left common carotid artery, and an artificial conduit was anastomosed. After re-establishing circulation, venous blood flow from the dissected area at the base of the aortic root was observed, indicating communication between the aorta and the right atrium. Circulatory arrest was induced again, and the ruptured outer aortic adventitia was repaired by continuous suturing using 5–0 prolene. The atrial fistula was repaired from within the right atrium using 5–0 prolene with felt reinforcement. Thus, successful closure was achieved. Proximal anastomosis and right brachiocephalic artery reconstruction were subsequently performed. Postoperative transesophageal echocardiography revealed no shunt flow and no bleeding from the aortic root. The patient recovered smoothly and was discharged without significant complications.

**Conclusions:**

Aorto-right atrial fistula associated with Stanford type A aortic dissection is rare, and in this case, the shunt blood flow was low, making preoperative diagnosis difficult. However, after intraoperative diagnosis, direct suture was used to complete the treatment, which was a simple and effective method.

## Background

When a Stanford type A aortic dissection develops and the aorta ruptures, it often perforates into the pericardial space. Herein, we report a rare case of aorto-right atrial fistula penetrating the right atrium in a patient with Stanford type A aortic dissection.

## Case presentation

**Fig. 1:**
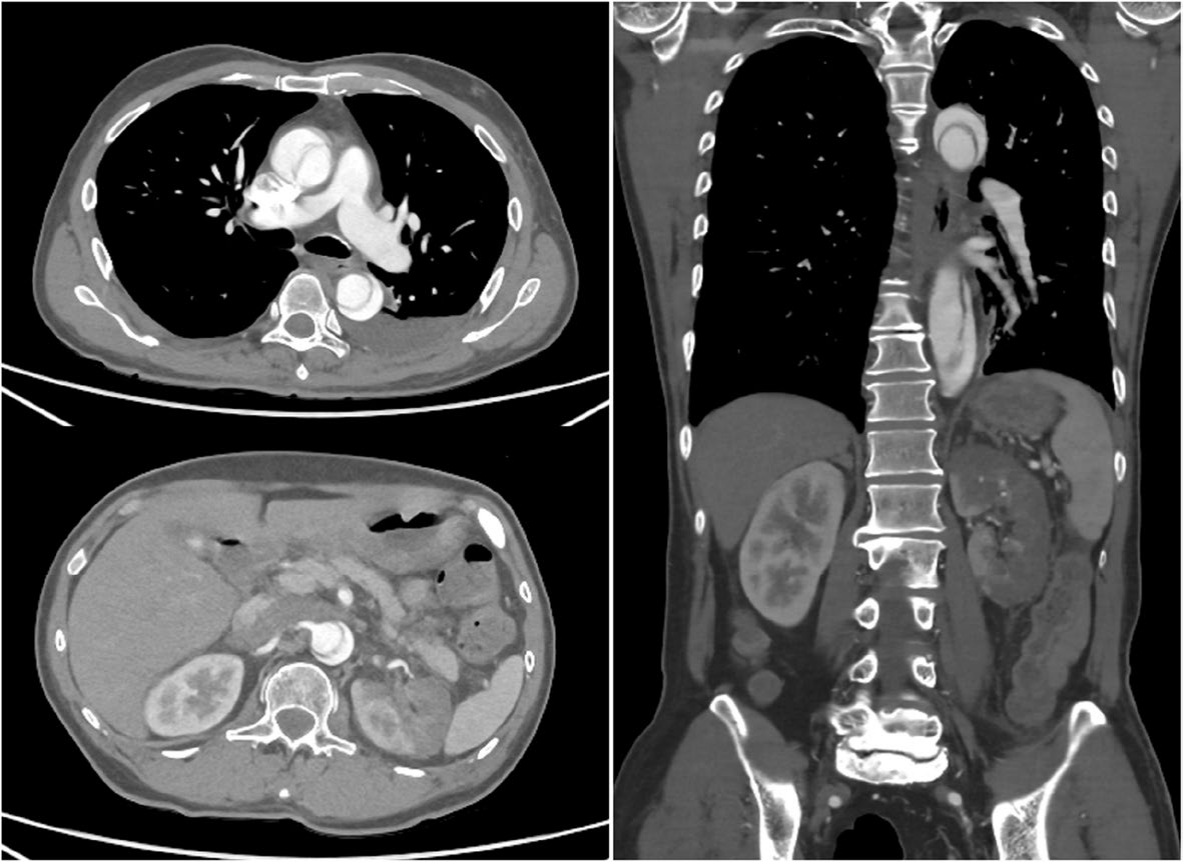
Preoperative contrast-enhanced computed tomography. The aortic dissection extends from the aortic root to the distal aorta. A left renal infarction is also present

A 60-year-old male with a history of rheumatoid arthritis treated with methotrexate experienced sudden lumbar pain accompanied by a fever that did not improve. After 2 days, the patient sought medical attention at our facility. Vital signs upon admission were as follows: blood pressure 143/65 mmHg, pulse rate 123/min, body temperature 39 °C, and SPO2 94%. Physical examination did not reveal significant abnormalities in cardiac or respiratory sounds, but tenderness during percussion of the left costovertebral angle was noted. Laboratory tests, including elevated white blood cell count (17,500/μL) and C-reactive protein (23.6 mg/dL), indicated inflammation. Elevated creatinine (1.9 mg/dL), creatine kinase (406 mg/dL), and lactate dehydrogenase (1579 IU/L) suggested impaired renal function and renal infarction. The patient was diagnosed with pyelonephritis and was admitted for further evaluation and treatment. The lumbar pain did not subside, even after initiating antibiotic therapy. Thus, a contrast-enhanced CT scan was performed on the following day. CT revealed Stanford type A aortic dissection extending from the aortic root to the distal aorta, with concurrent partial right renal infarction (Fig. 1). Emergency surgery was scheduled for the same day.

**Fig. 2:**
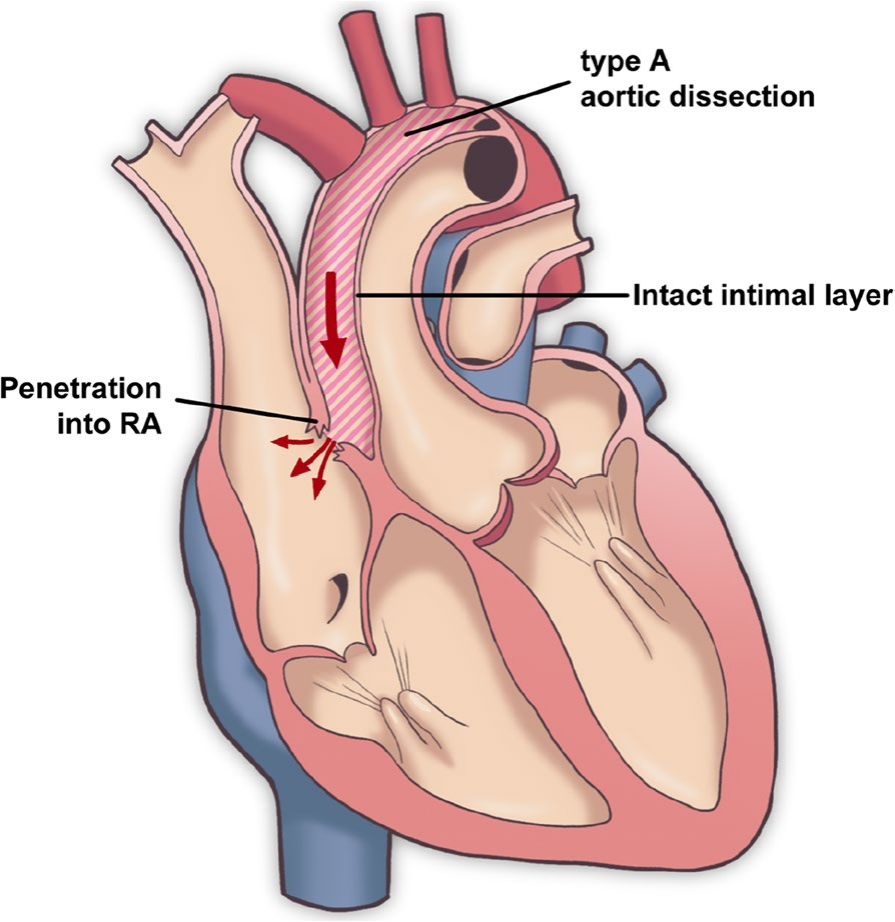
Schema of the false lumen in the aorta and the fistula between the aorta and right atrium. The aortic intima remained intact while the false lumen had ruptured

**Fig. 3:**
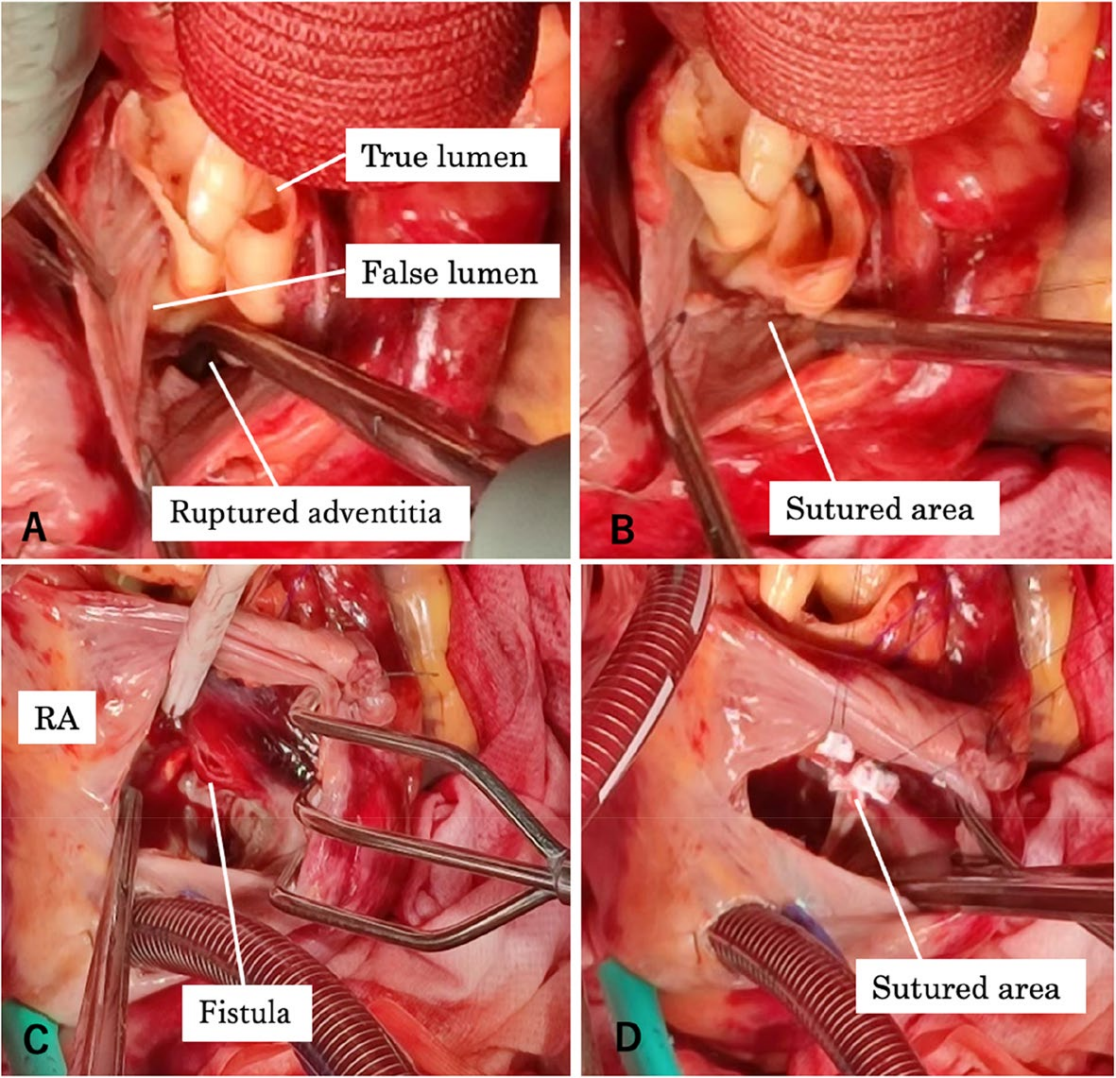
**A** Intraoperative image showing the ruptured adventitia. **B** Intraoperative image depicting the closure of the adventitia using continuous sutures with 5–0 prolene. **C** Intraoperative image showing the fistula from within the right atrium. **D** Intraoperative image showing closure using mattress sutures with 5–0 prolene and felt

Femoral arterial inflow, right atrial drainage, and left atrial venting for cardiopulmonary bypass were established. The patient was cooled from cardiopulmonary bypass and prepared for circulatory arrest. Initially, during intraoperative TEE, attention was not directed toward the presence of an aortic-right atrial fistula. After reaching 29 °C body temperature, circulatory arrest was instituted, and the aorta was incised to induce selective cerebral perfusion. After inspecting the aorta, a primary entry was identified on the anterior surface of the ascending aorta. The aorta was dissected between the right brachiocephalic artery and the left common carotid artery, and then Feltect (Kono Seisakusho Co., Ltd., Tokyo, Japan) was placed inside and outside the aorta, and transection was performed with a felt sandwich. Then, a 24-mm J-graft (Japan Lifeline Co., Ltd., Tokyo, Japan) was anastomosed at the point of aortic interruption using 4–0 prolene. When circulation was resumed, venous blood flow was noted from the dissection site at the aortic root. Intravascular examination of the aorta revealed no abnormalities in the Valsalva sinus or aortic valve. However, a crack was identified in the adventitia of the false lumen, corresponding to the non-coronary cusp of the aortic valve. This crack led to a rupture into the right atrium (Fig. 2). Consequently, the circulatory arrest was reinstituted, and the surgical approach was modified to drain the right atrium and both superior and inferior venae cavae. The rupture site in the adventitia was closed using continuous suturing with 5–0 prolene. The crack in the right atrium was approximately 8 mm and was located near the anterior cusp of the tricuspid valve. The rupture site in the right atrium was closed with 5–0 prolene sutures reinforced with felt (Fig. 3). The false lumen at the aortic root was then crimped with BioGlue (CryoLife Inc., Kennesaw, GA, USA), Feltect (Kono Seisakusho Co., Ltd., Tokyo, Japan) was placed inside and outside the aorta, reinforced with a felt sandwich, and transection was performed. Proximal anastomosis and reconstruction of the right brachiocephalic artery were performed. After restoration of the patient’s heart rhythm, TEE revealed no shunt flow, and no bleeding was observed from the root. The duration of cardiopulmonary bypass was 212 min, cardiac arrest lasted 136 min, and circulatory arrest lasted 50 min.

The patient recovered without significant complications. Postoperative transthoracic echocardiogram revealed no residual shunt flow. Contrast-enhanced CT imaging was not feasible due to renal impairment, but non-contrast CT did not reveal any significant abnormalities in the aorta or heart. The patient was discharged on the 26th postoperative day and was followed up in the outpatient clinic. The patient is experiencing a satisfactory recovery two years post-surgery.

## Discussion

Ruptures into the pericardial or pleural cavity are common in patients with Stanford type A aortic dissections. However, rupture into the right atrium, which was initially reported by Kuipers and Schatz in 1963, is exceedingly rare [1]. Rupture into the right atrium is usually diagnosed postmortem, but Temple and colleagues reported the first successful surgical treatment of aortic dissection complicated by aortic-right atrial fistula [2]. Rupture into the right atrium can exacerbate heart failure due to increased load on the right heart system, potentially leading to a fatal outcome.

Preoperative diagnosis of aortic-right atrial fistula is challenging but can be achieved with TEE showing shunt flow from the aorta to the right atrium [3]. However, in our case, preoperative diagnosis of the aortic-right atrial fistula using TEE was not possible, likely due to the subtle symptoms and delayed manifestations of right heart failure even 2 days after onset. Therefore, when performing surgery for type A acute aortic dissection, it is necessary to consider the possibility of this disease even if it cannot be observed by preoperative TEE.

Repair techniques for right atrial fistulae include aortic root replacement [3], closure using pericardial or Dacron patches [4], and direct closure by suturing [5]. While aortic root replacement is the most reliable repair method, this technique is more invasive than other procedures. Closure using pericardial or Dacron patches is an effective treatment when preoperative diagnosis is made through TEE, allowing for adequate preoperative preparation. In cases where preoperative diagnosis of aortic-right atrial fistula is not possible, as in our case, immediate use of patches can be challenging without prior preparation. In our case, the rupture of the adventitia was in a location amenable to direct closure; thus, the rupture was repaired with direct sutures. Closure from the right atrial side was also achieved through direct sutures from within the atrium, effectively closing the fistula. No residual shunt flow was observed postoperatively, and closure through direct sutures proved to be an effective treatment.

In conclusion, we experienced a rare aorto-right atrial fistula resulting from Stanford type A aortic dissection. Although the shunt blood flow was low and preoperative diagnosis was difficult, the treatment could be completed by direct suture, which was a simple and effective surgical method.

## Data Availability

All data supporting the conclusions of this article are included within the published article.

## Abbreviations

CT: Computed tomography
TEE: Transesophageal echocardiography
RA: Right atrium
